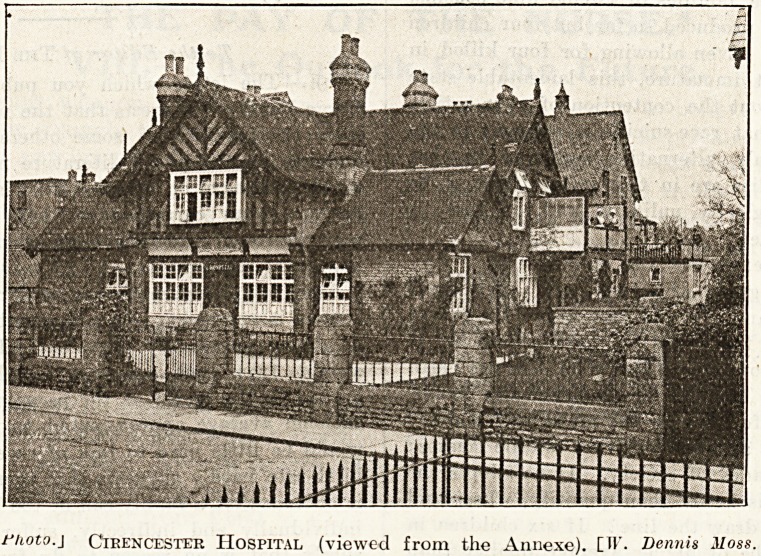# A Paying Hospital for All Classes and All Incomes

**Published:** 1921-04-30

**Authors:** 


### THE CIRENCESTER EXPERIMENT.

### A Paying Hospital for all Classes and all Incomes.

The Committee of the Cirencester Hospital have satisfactorily solved the problem of voluntaryism. They have put into practice a scheme evolved by one of the medical men of the town which effects a working compromise between the purely charitable form of voluntaryism designed to assist only the necessitous poor, and the modern Continental method of supplying first-class institutional treatment at a moderate price to the middle-classes. They have enlisted into their scheme all the medical men of the neighbourhood; they have adequately discharged their obligations to the poor of the town; and have so successfully won the confidence of the general practitioners' private patients that the doctors recommend them to go to the local hospital for operative or other special treatment—and the patients go. Yet the Committee has succeeded in enlisting a wider public than ever in the work of financially supporting the institution, and it pays its way.

In the first series of Reports on Hospitals of the United Kingdom the late Sir Henry Burdett described the excellent work done by this one of the most interesting cottage hospitals in the world (The Hospital, November 13, 1909, p. 188). The hospital itself was founded in 1875 by the late Mr. Allen Bathurst, M.P., afterwards sixth Earl Bathurst, for the benefit of the poor of the town and neighbouring parishes. For every guinea subscribed annually supporters of the hospital had, and still have, the right to recommend patients for admission. Thus for years the institution was financed in the good old way by the generous subscriptions of the gentry and traders of the neighbourhood.

*Photo.*] Cirencester Hospital (viewed from the Annexe). [*W. Dennis Moss.*

The war made changes at Cirencester as elsewhere, and this hospital felt the pinch of the new poverty pressing on the upper and middle classes. To earn money for its support and to meet the needs of the "black-coat" workers and new poor, it was decided that private paying wards should be started, and that all patients must be asked to pay, according to their means, for treatment. The accommodation originally provided for the poor of the town could not be encroached upon for the private patients. So the nurses' quarters were taken and transformed into private single and two-bedded wards, and as the nurses and the $x$-ray and electrical equipment now had to be housed somewhere, the Cirencester people decided to provide them with an abiding place as the town's memorial to the fallen. Lord Bathurst offered the Memorial Committee a hall just across the road from the hospital, and this the honorary architect was able to reconstruct, at considerable saving of expense, as a hospital annexe and nurses' hostel. This annexe consists, on the ground floor, of an up-to-date $x$-ray department, with adjoining dark room, a pathological laboratory, an electrical treatment room, a massage and remedial exercises room, dressing rooms, waiting room, and doctors' consulting room. The electrical department is lavishly equipped with all kinds of baths, nerve-testing, and muscle-re-educating apparatus; and the mechano-therapy room and laboratory are also well stocked and supplied. In the basement is a suite of rooms for the resident radiographer-pathologist, store-rooms, etc., and above is one of the most modern and comfortably furnished nurses' hostels that even a member of the College of Nursing could wish to see.

The sceptical administrative officer will be asking, "But will it pay the hospital?" While the general practitioner may be murmuring, "Will it pay the doctor in private general practice?" The experience of Cirencester over a period of two years gives an emphatic affirmative by way of answer. In 1920 expenditure for the year amounted to £2,604 odd, including over £231 for structural improvements and new equipment. Receipts from all sources totalled £2,548 odd, but there was a balance of £56 in hand with which to begin the year, so that the small deficit was covered. Of these receipts ordinary patients' fees accounted for £334, private patients' fees for £613, War Pensions Committee patients for £33, and $x$-ray and massage patients for £184, over £1,160 in all. Subscriptions have been well maintained. The explanation is that an increasing number of private patients are taking advantage of the scheme, and that people of all classes, knowing that they themselves may one day benefit by treatment at the hospital, are coming forward to support it. Particularly valuable sources of financial support are the contributions from organised associations of workers and traders, who realise that in assisting this institution they are quite possibly investing their money in something that may one day stand between themselves and the risk of death, and in any case will prove of benefit to some of their associates. As even the ordinary poor patients now pay not less than 1s. per day in fees, all classes feel that they are paying their way to the best of their ability. An excellent feature of the Cirencester scheme is that the Town Nurse works in connection with the hospital and under its medical officers, so that patients discharged from the institution can be followed up by the Town Nurse without any break in the continuity of the medical advice or the nursing.

Private patients must present a guarantee, signed by themselves or their friends, that they will ensure the weekly payment of any sum assessed by the Committee, which will not be less than £2 2s. per week, exclusive of the cost of extra nursing, dressings, special appliances, and medicines, and the application for admission which bears the guarantee must be endorsed by their medical attendant. The amount all patients are required to pay is assessed before admission by a special committee representative of all classes of the community, at whose sitting the applicant's medical man is required to be present in the capacity of applicant's advocate. In the assessment book are to be seen entries of assessments varying from 9d. per diem to £10 10s. per week.

The medical men of the neighbourhood are among the most enthusiastic supporters of the scheme. The very existence of such a local institution must prove an attraction to skilled surgeons to practise in the district. The Cirencester general practitioners have not been slow to realise that the hospital gives them added opportunities of studying interesting cases and keeping themselves clinically abreast of modern discoveries and improved methods of diagnosis and treatment. The pathological laboratory, the $x$-rays, and the electrical testing apparatus are proving priceless boons to all these men in their routine practice. None of them is resident at the hospital, but each takes turns to be honorary orderly officer for a month at a stretch. During this period of duty the medical man in charge takes on all emergency cases that are admitted, and attends to all his colleagues' cases if occasion arise and these doctors for any reason cannot be summoned to the hospital within a reasonable time. He also acts as medical superintendent, and as such is responsible for the smooth running of the hospital during his duty month, giving a report to the Committee and the other medical officers at the close of his stewardship. The system, thanks, no doubt, to the good-will of the medical men and the matron alike, runs without friction.

## Figures and Tables

**Figure f1:**